# Construction of a High-Precision Underwater 3D Reconstruction System Based on Line Laser

**DOI:** 10.3390/s25185615

**Published:** 2025-09-09

**Authors:** Hongyang Xu, Yongqing Zeng, Mingyu Qiu, Xiaoping Wang

**Affiliations:** 1 Ocean College, Zhejiang University, Zhoushan 316000, China; 22334034@zju.edu.cn (H.X.); 22434036@zju.edu.cn (Y.Z.); 22434009@zju.edu.cn (M.Q.); 2 Donghai Laboratory, Zhoushan 316000, China

**Keywords:** line laser rotation scanning, joint calibration of light plane and rotation axis, refraction model compensation, centerline extraction

## Abstract

This study presents a high-precision underwater 3D reconstruction system based on rotating laser scanning. The system adopts a modeling framework that combines air-based camera calibration with refraction compensation. A five-stage image preprocessing pipeline, followed by an adaptive locally weighted centroid (ALWC) algorithm, is employed to extract the laser stripe centerline with high accuracy. In addition, a synchronized feature extraction-based joint calibration method is introduced to simultaneously calibrate the light plane and the rotation axis. Resolution evaluation experiments demonstrate that the system achieves a spatial resolution of better than 0.6 mm in clear water at a distance of 1 m, and approximately 0.8 mm in turbid water conditions of 6 Nephelometric Turbidity Units (NTU), thereby verifying its reconstruction accuracy and robustness under varying water clarity.

## 1. Introduction

In recent years, the underwater 3D optical measuring system, as an active optical measurement means, has demonstrated significant application potential in complex marine environments by virtue of its unique high resolution and strong anti-interference capability [1]. Different from the traditional sonar system relying on the acoustic wave propagation mechanism, laser scanning has stronger directionality and spatial resolution by actively emitting a high-energy narrow-beam laser and is based on the principle of optical triangulation, which can realize high-precision modeling of complex underwater structures and provide reliable technical support for refined underwater detection [2,3,4].

This technology has been widely applied across multiple domains. In marine science, it enables the acquisition of key information such as seafloor mineral deposits [5] and subsea oil and gas distributions [6], supporting detailed geomorphological modeling of the seabed [7]. In underwater engineering inspection, it facilitates structural integrity assessments of dams, hydropower stations, submarine pipelines, and other critical infrastructure [8]. Moreover, in scenarios involving underwater object recognition and marine life observation, 3D laser imaging also supports high-accuracy modeling of complex underwater structures [2,3,4]. Furthermore, 3D laser imaging supports robotic navigation, structural recognition, and quantitative analysis of biological morphology in underwater target detection and marine biology applications [9,10,11].

The underwater laser scanning system integrates advanced technologies from optical imaging, image processing, computer vision, and point cloud analysis to achieve high accuracy and robustness. Typically, the system comprises a scanning module—consisting of a line laser and a high-speed camera—that acquires images via structured laser illumination, combined with rotational or translational mechanisms to enable multi-angle data acquisition [12,13]. The subsequent data processing workflow involves essential steps such as camera calibration, optical plane modeling, laser stripe centerline extraction, and point cloud registration, which must be executed sequentially to ensure reconstruction accuracy [14].

Mansbach [15], building on the pinhole imaging model and triangulation principles, established the spatial relationship between the camera and the laser plane by calibrating the laser projection angle and baseline distance, subsequently deriving a coordinate transformation model. Tsai [16] proposed a classical calibration method based on radial constraints, while Zhang Z [17] developed a widely adopted single-response matrix model utilizing tessellated grid patterns. Chen [18] further optimized the calibration pattern by employing circular structures and enhanced feature point detection accuracy through ellipse fitting techniques. Xu G [19] introduced the Plücker coordinate system to solve laser plane equations, improving model stability. Later, Zhang Z [20] and Li T [21] refined the rotating-axis estimation process, enhancing spatial modeling precision in structured light systems.

Following system calibration, precise extraction of the laser stripe center in captured images becomes a critical factor influencing 3D reconstruction accuracy [22]. Extraction methods primarily fall into three categories: grayscale-based techniques (e.g., maximum value method, center of gravity method [23]), gradient-based approaches leveraging Hessian matrices such as Steger’s algorithm [24], and intelligent methods incorporating deep learning with image priors [25].

To improve laser stripe extraction accuracy in underwater environments, various preprocessing techniques—such as spatio-temporal filtering, morphological enhancement, and deep neural networks—have been introduced recently. For instance, Ye T [25] enhanced laser stripe response by combining adaptive scale kernel functions, while Tran [26] proposed a point cloud splicing approach based on implicit volume modeling, effectively mitigating occlusion and matching errors.

In the context of point cloud splicing, accurate coordinate alignment across different viewpoints and temporal frames is crucial. Lin H [27] developed a reconstruction method leveraging multi-parameter calibration and aberration correction to achieve reliable results in highly turbid waters. Researchers such as Lopes [28] and Palomer [29] attained millimeter-level reconstruction accuracy by constructing dual-laser systems or employing high-frequency acquisition devices, all while maintaining system lightweightness. Additionally, Bleier’s team [30] developed a 525 nm underwater laser that preserves imaging quality in dynamic water environments. Wang et al. [31] enhanced calibration robustness and image analysis through deep neural networks.

Despite these advances, underwater optical attenuation, refractive distortion, and random noise remain major obstacles limiting current system accuracy. Istenič [32] proposed a laser scaling-based method to enhance accuracy, while Halimi et al. [33] applied a hierarchical Bayesian framework to improve 3D reconstruction under high noise. Castillon [34] introduced a biaxial mirror-based underwater imaging system that geometrically converts curved laser paths to straight lines, effectively reducing refraction-induced distortions.

Underwater line-laser scanning systems face several intrinsic challenges. First, suspended particles and turbidity fluctuations induce strong scattering and absorption, degrading image quality. Second, the propagation of laser and imaging light across multiple media interfaces results in complex refraction phenomena, causing geometric distortions. Third, the reflectance properties of target surfaces—determined by material composition, surface roughness, and incident angles—affect laser intensity distribution in captured images, directly impacting the quality of resulting 3D point clouds.

In this study, we propose an integrated underwater laser scanning system and 3D reconstruction framework that encompasses the entire pipeline—from data acquisition and image processing to geometric modeling and point cloud enhancement. The main contributions are as follows: (1) implementation of modular control based on an embedded platform; (2) development of a joint calibration method for the laser plane and rotation axis using single-frame acquisition, improving system geometric consistency; (3) construction of a robust centerline extraction algorithm tailored to low-turbidity water, enhancing laser stripe detection and localization accuracy; (4) establishment of a physical multi-interface refraction model to correct reconstruction errors caused by light deviation; and (5) proposal of a reflectivity-aware point cloud compensation strategy to improve surface detail fidelity in the reconstructed slices. The system has been validated in 6 NTU turbidity conditions, demonstrating strong resilience to multiple interferences while maintaining high spatial resolution.

This paper is organized as follows: Section 2 introduces the composition and operating principles of the underwater laser scanning system. Section 3 details the system parameterization methods for camera calibration, laser plane modeling, rotation axis estimation, and refraction compensation. Section 4 presents an adaptive locally weighted centroid (ALWC) algorithm with five-step preprocessing, consistency check, and interpolation for robust underwater laser stripes. Section 5 presents 3D reconstruction results and error analyses. Finally, Section 6 concludes the study and discusses future research directions.

## 2. Components and Structure of an Underwater Laser Scanning System

**Figure 1:**
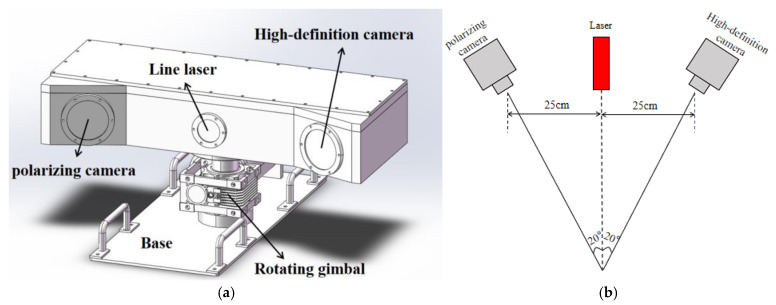
Underwater laser scanning system. (**a**) 3D model of the prototype; (**b**) camera–laser triangular structural diagram with dimensions.

The system employs an active rotary scanning strategy, replacing the conventional linear sliding method. This approach effectively addresses the limitations of restricted measurement range and structural redundancy caused by the finite length of the sliding rail. The compact, all-in-one design significantly enhances the system’s adaptability and deployability in real underwater environments, as illustrated in Figure 1a.

The system integrates multiple modules, including a line laser, high-definition camera, polarization camera, rotating gimbal, and embedded control unit, enabling autonomous rotation and synchronized data acquisition. Specifically, the laser source is a 640 nm uniform line laser with an output power of 150 milliwatts, providing stable and high-intensity illumination for underwater scanning. The imaging module employs a 12-megapixel industrial area-scan camera based on a 1.1-inch CMOS sensor with a USB 3.0 interface, ensuring high-resolution and high-speed image capture. The entire device is compactly designed with dimensions of 500 mm × 200 mm × 240 mm (length × width × height) and a weight of approximately 10 kg in air, which facilitates convenient deployment and stable operation in real underwater environments.

As shown in Figure 1b, the system’s camera and laser are arranged in a triangular configuration. The distance between each camera and the laser is 25 cm, and the angle between the camera’s optical axis and the laser projection direction is 20°, effectively balancing measurement accuracy and system compactness. The system achieves omnidirectional scanning without relying on a large underwater platform.

**Figure 2:**
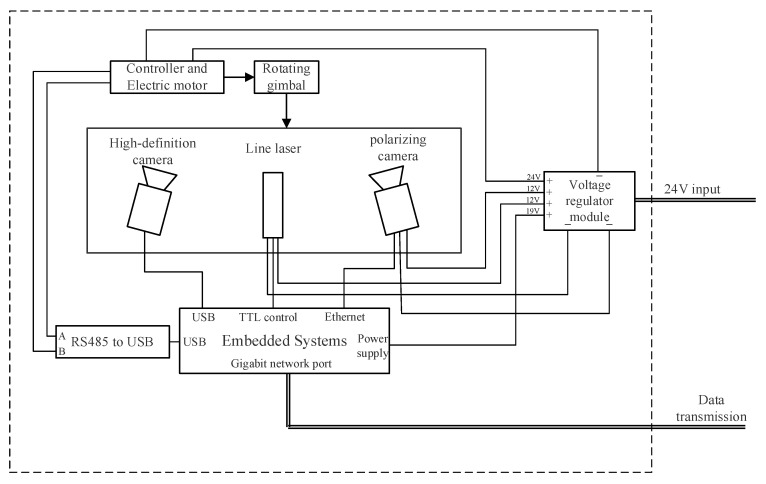
Schematic diagram of underwater encapsulation.

Figure 2 illustrates the underwater housing structure and module connection logic of the system. The system integrates a high-definition camera, polarization camera, line laser, and rotary head, achieving high-precision rotational control via a stepper motor. All modules are coordinated by an embedded system to perform image acquisition, centerline extraction, and data processing. The housing features excellent waterproof sealing and high integration, ensuring long-term stable operation underwater. Additionally, the system includes a reserved interface for the polarization camera, providing scalability for future implementation of polarization-difference-enhanced imaging in medium to high turbidity environments.

**Figure 3:**
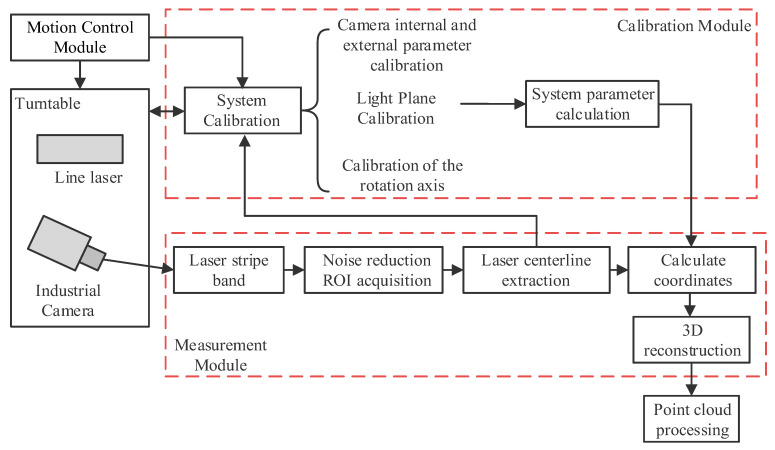
Overall technical route.

Figure 3 presents the overall architecture and functional workflow of the underwater laser 3D scanning system developed in this study. The motion control module drives the rotary stage via a motor to enable omnidirectional scanning of the laser stripe across the target object. The calibration module performs joint calibration of the camera’s intrinsic and extrinsic parameters, the laser light plane, and the rotation axis, thereby computing system parameters to provide an accurate geometric foundation for 3D coordinate reconstruction. The measurement module first extracts laser stripes from the captured images. Following dynamic region of interest (ROI)-based noise suppression, the centerline is extracted using an adaptive locally weighted centroid (ALWC) method. High-density point cloud data are then generated to complete the 3D reconstruction.

## 3. Determine System Parameters

The operating principle of a line laser scanning system is based on detecting the line of intersection between a laser plane and the surface of the target object. A camera captures the projection of this intersection line from different positions. The geometric relationships among the camera, the laser plane, and the rotation axis determine how pixel information extracted from images is mapped into three-dimensional space. Accurate calibration of the camera’s intrinsic and extrinsic parameters, the laser plane equations, and the position and orientation of the rotation axis forms the foundation for building a precise geometric model.

### 3.1. Calibration of Intrinsic and Extrinsic Parameters of the Camera

In this study, Zhang’s calibration method [17] is adopted, using a 7 × 7 circular dot-matrix calibration plate as the target.

**Figure 4:**
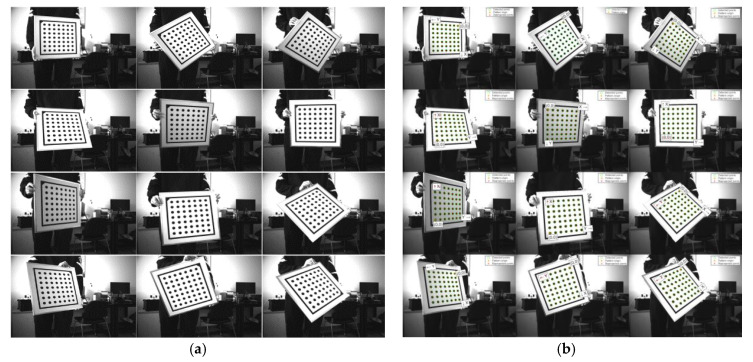
Camera calibration process. (**a**) Shots of circular calibration plates at different angles; (**b**) feature point extraction results.

Compared with traditional checkerboard calibration targets, circular dot patterns demonstrate greater robustness under conditions of edge blur, slight defocus, or poor contrast. During the experiment, the camera remains fixed while the calibration plate is manually translated and rotated to present various poses and viewpoints. As shown in Figure 4, the system captures 12 grayscale images of the calibration plate, covering diverse regions and orientations within the image plane.

We can transform the pixel coordinates $[u,v{]}^{T}$ to the 3D coordinates $[X_{w},Y_{w},Z_{w}{]}^{T}$ in the world coordinate system, and the complete coordinate transformation formula is [17]

(1) $$Z_{c}\left[\begin{array}{c} u\\ v\\ 1 \end{array}\right]=\left[\begin{array}{cccc} f_{x} & 0 & c_{x} & 0\\ 0 & f_{y} & c_{y} & 0\\ 0 & 0 & 1 & 0 \end{array}\right]\left[\begin{array}{cc} R & t\\ 0 & 1 \end{array}\right]\left[\begin{array}{c} X_{w}\\ Y_{w}\\ Z_{w}\\ 1 \end{array}\right]$$

where $Z_{c}$ is the depth component in the camera coordinate system and R, t are the camera extrinsic parameters.

Optical distortions often arise during image formation due to lens design and imaging principles, impacting spatial geometric accuracy. The distortion correction model is given by [17]

(2) $$\left[\begin{array}{c} x_{\mathrm{corrected}\;}\\ y_{\mathrm{corrected}\;} \end{array}\right]=\left(1+k_{1}r^{2}+k_{2}r^{4}+k_{3}r^{6}\right)\left[\begin{array}{c} x\\ y \end{array}\right]+\left[\begin{array}{c} 2p_{1}xy+p_{2}\left(r^{2}+2x^{2}\right)\\ 2p_{2}xy+p_{1}\left(r^{2}+2y^{2}\right) \end{array}\right]$$

where $[x,y{]}^{T}$ are the coordinates of the uncorrected point, ${\left[x_{\mathrm{corrected}}\;,y_{\mathrm{corrected}}\right]}^{T}$ are the coordinates of the corrected point, $r=\sqrt{x^{2}+y^{2}}$ is the distance of the point from the imaging center, $k_{1}$, $k_{2}$, $k_{3}$ are the radial aberration coefficients, and $p_{1}$, $p_{2}$ are the tangential aberration coefficients.

**Figure 5:**
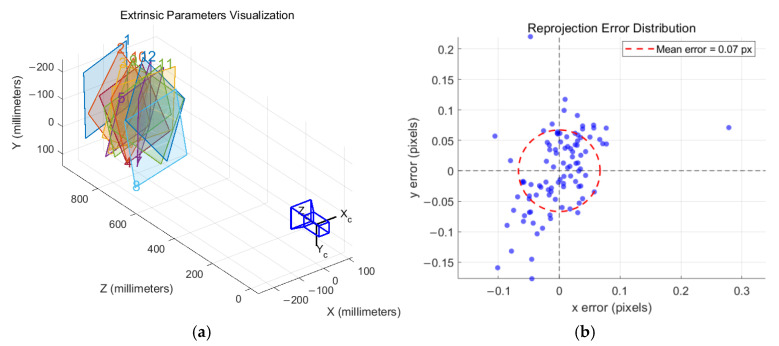
Outer parameter visualization results and reprojection error distribution. (**a**) Outer parameter visualization results; (**b**) reprojection error distribution for the calibration camera.

In this study, the camera is equipped with an MVL-KF1228M-12MP industrial fixed-focus lens manufactured by HIKROBOT in Hangzhou, China. The lens has a focal length of 12 mm. It exhibits high manufacturing precision with negligible tangential distortion. Therefore, only radial distortion was modeled and corrected. The final calibration yields an average reprojection error of approximately 0.07 pixels (Figure 5b), indicating high accuracy and stability of the calibration.

### 3.2. Construction of an Underwater Refraction Model

Due to the complexity, low image quality, and instability often associated with direct underwater calibration, this study adopts an “air calibration + refraction model compensation” strategy for system geometric modeling. First, a circular calibration plate is used to calibrate the camera’s intrinsic parameters in air, yielding a high-precision pinhole model. Then, a physical refraction model is applied to correct the direction of each pixel under underwater conditions. Finally, a nonlinear mapping function is employed to perform the inverse projection from image pixels to 3D coordinates in the underwater scene.

In the underwater laser 3D scanning system, the observed positions of laser stripes in images correspond to apparent coordinates caused by refraction at the air–glass–water interfaces, rather than the true 3D spatial coordinates of the target surface. Direct use of the traditional pinhole camera model for triangulation leads to systematic offsets and scale errors in 3D reconstruction.

In underwater environments, light refracts at the interface between different media, such as air and water, in accordance with Snell’s law. Although refraction occurs both at the air–glass and glass–water interfaces, the light path inside the glass window can be approximated as a straight line due to the window’s minimal thickness and parallel surfaces. This study’s experimental setup uses a thin quartz glass watertight window in a laboratory environment; therefore, to simplify modeling and improve computational efficiency, the refractive effect of the glass medium is neglected. Consequently, the underwater imaging model is simplified to consider only single refraction at the air–water interface.

Let $n_{\mathrm{water}}$ and $n_{\mathrm{air}}$ be the refractive indices of water and air, respectively, and $d_{w}={\left[d_{wx},d_{wy},d_{wz}\right]}^{T}$ and $d_{a}={\left[d_{ax},d_{ay},d_{az}\right]}^{T}$ represent the direction vectors of the incident light before and after refraction, according to the projection relationship between the unit direction vectors and Snell’s law; the direction vectors after refraction can be expressed as follows:

(3) $$\left[\begin{array}{c} d_{wx}\\ d_{wy}\\ d_{wz} \end{array}\right]=\frac{n_{\mathrm{air}}}{n_{\mathrm{water}}}\left[\begin{array}{c} d_{ax}\\ d_{ay}\\ \sqrt{\frac{n_{\mathrm{water}}^{2}}{n_{\mathrm{air}}^{2}}+d_{az}^{2}-1} \end{array}\right]$$

This is known from the geometric relationship:

(4) $$\left[\begin{array}{c} d_{ax}\\ d_{ay}\\ d_{az} \end{array}\right]=\frac{1}{\sqrt{x_{i}^{2}+y_{i}^{2}+f^{2}}}\left[\begin{array}{c} x_{i}\\ y_{i}\\ f \end{array}\right]$$

where f is the focal length of the camera and $[x_{i},y_{i}{]}^{T}$ are the 2D image coordinates of the imaging point.

Let $r=\sqrt{x_{i}^{2}+y_{i}^{2}+f^{2}}$, and the ratio of refractive index $n_{0}=n_{\mathrm{water}}/n_{\mathrm{air}}$; the relationship between the direction vector of the incident ray and the image coordinates can be found as

(5) $$\left[\begin{array}{c} d_{wx}\\ d_{wy}\\ d_{wz} \end{array}\right]=\frac{1}{n_{0}}\left[\begin{array}{ccc} \frac{1}{r} & 0 & 0\\ 0 & \frac{1}{r} & 0\\ 0 & 0 & \sqrt{n_{0}^{2}+{\left(\frac{f}{r}\right)}^{2}-1} \end{array}\right]\left[\begin{array}{c} x_{i}\\ y_{i}\\ 1 \end{array}\right]$$

In order to realize the mapping from the image pixel coordinates to the target point spatial coordinates, it is necessary to introduce the geometric relationship between the direction of the refracted light ray and the refraction plane for modeling. Assuming that D represents the distance between the refraction plane and the center of the camera, ${\left[x_{p},y_{p},z_{p}\right]}^{T}$ are the coordinates of the intersection point of the light ray and the refraction plane in the camera coordinate system, and ${\left[x_{c},y_{c},z_{c}\right]}^{T}$ are the three-dimensional coordinates of the target point in the camera coordinate system; then,

(6) $$k\left[\begin{array}{c} d_{wx}\\ d_{wy}\\ d_{wz} \end{array}\right]=\left[\begin{array}{llll} 1 & 0 & 0 & -\frac{x_{i}D}{f}\\ 0 & 1 & 0 & -\frac{y_{i}D}{f}\\ 0 & 0 & 1 & -D \end{array}\right]\left[\begin{array}{c} x_{c}\\ y_{c}\\ z_{c}\\ 1 \end{array}\right]$$

where k is the scale factor of the target point in the refraction direction, reflecting the geometric depth relationship between the incident direction and the actual spatial point.

Further, to compensate for the nonlinear aberration due to underwater refraction, the pixel coordinates need to be directionally corrected. According to the refraction modeling formula, in the case of a known pixel point $[u,v{]}^{T}$, the scale factor k corresponding to its refraction direction can be approximated as

(7) $$k\approx \frac{\tan\left(\arcsin\left(\frac{\sin\left(\arctan\frac{\sqrt{u^{2}+v^{2}}}{f}\right)}{n_{\mathrm{water}}}\right)\right)}{\frac{\sqrt{u^{2}+v^{2}}}{f}}$$

The relationship between the coordinates of the object in the camera coordinate system and the coordinates in the image coordinate system can be obtained as

(8) $$k\left[\begin{array}{c} x_{i}\\ y_{i}\\ 1 \end{array}\right]=n_{0}{\left[\begin{array}{ccc} \frac{1}{r} & 0 & 0\\ 0 & \frac{1}{r} & 0\\ 0 & 0 & \sqrt{n_{0}^{2}+{\left(\frac{f}{r}\right)}^{2}-1} \end{array}\right]}^{-1}\left[\begin{array}{llll} 1 & 0 & 0 & -\frac{x_{i}D}{f}\\ 0 & 1 & 0 & -\frac{y_{i}D}{f}\\ 0 & 0 & 1 & -D \end{array}\right]\left[\begin{array}{c} x_{c}\\ y_{c}\\ z_{c}\\ 1 \end{array}\right]$$

where $r=\sqrt{x_{i}^{2}+y_{i}^{2}+f^{2}}$, $n_{0}=n_{\mathrm{water}}/n_{\mathrm{air}}$.

**Figure 6:**
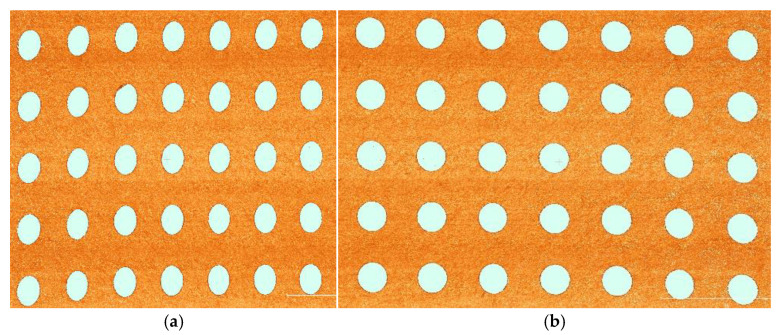
Example of point cloud before and after refraction correction: (**a**) before refraction correction; (**b**) after refraction correction.

By retaining the high calibration precision achieved via in-air calibration and integrating nonlinear refraction correction derived from Snell’s law, we establish a complete geometric mapping framework that converts 2D pixel coordinates to 3D spatial coordinates. The effectiveness of this refraction correction is clearly demonstrated in Figure 6, which presents 3D point clouds of the scanned circular calibration plate. Figure 6a shows the point cloud prior to refraction correction, with noticeable geometric distortion induced by light refraction at the air–water interface; in contrast, Figure 6b displays the optimized point cloud after correction, where the contour of the circular calibration plate is more consistent with its actual physical shape, directly verifying the reduction of refraction-induced errors.

### 3.3. Joint Optical Plane-Rotation Axis Calibration Method Based on Synchronized Feature Extraction

Traditional calibration approaches typically estimate the spatial positions of the optical plane and the rotation axis separately by acquiring images or conducting different experimental steps independently. This sequential procedure not only complicates the operation but also causes error accumulation across independent stages, adversely affecting the final geometric modeling accuracy.

**Figure 7:**
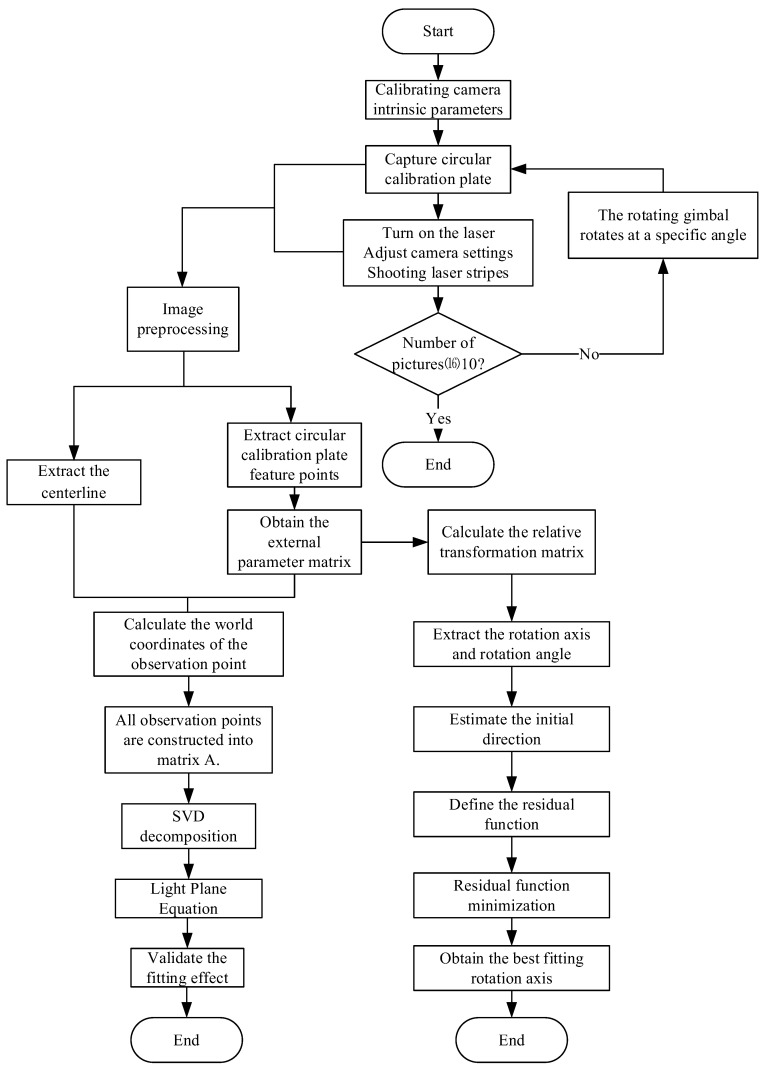
Workflow of the joint calibration process for the optical plane and rotation axis.

To address these issues, this study proposes a calibration method for the optical plane and rotation axis using the same synchronized image dataset. Laser stripe features and camera pose information are extracted from the same images to ensure consistent feature correspondence. The optical plane is robustly fitted using singular value decomposition (SVD); the rotation axis direction is estimated via nonlinear least squares optimization; and the rotation center is determined by linear fitting of the circular trajectory. The overall workflow is illustrated in Figure 7.

**Figure 8:**
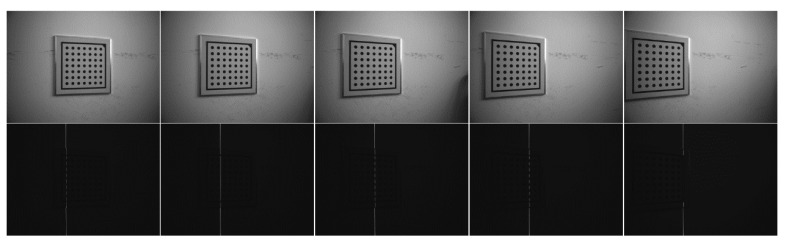
Experimental procedure of joint calibration of optical plane and rotation axis.

For accurate modeling of the laser plane, a joint calibration procedure controlled by the rotary head is designed as follows: the system sequentially rotates the rotary head to multiple discrete angles. At each angle, two types of images are captured: (1) images of the circular calibration plate without laser projection, used for estimating the spatial position of the plate; and (2) images of the projected laser stripes, used for laser centerline extraction, as shown in Figure 8.

To improve the stability and accuracy of centerline extraction in images containing laser stripes, the camera exposure time is suitably reduced to enhance the contrast between the laser stripes and the background. After extracting pixel coordinates with the centerline algorithm, these points are first back-projected into the world coordinate system based on the calibration plate’s extrinsic matrix, then uniformly transformed into the camera coordinate system to obtain their 3D spatial positions. Finally, SVD is applied to fit an optimal plane that minimizes the sum of squared distances from all points to the plane.

Let the world coordinates of the feature point be ${\left[X_{w},Y_{w},0\right]}^{T}$, the pixel coordinates be ${\left[u,v\right]}^{T}$, and the camera transformation matrix be

(9) $$\left[R|t\right]=\left[\begin{array}{llll} R_{11} & R_{12} & R_{13} & t_{x}\\ R_{21} & R_{22} & R_{23} & t_{y}\\ R_{31} & R_{32} & R_{33} & t_{z} \end{array}\right]$$

Transforming the points in the world coordinate system to the camera coordinate system by means of an extrinsic matrix, and subsequently projecting the points in the camera coordinate system to the pixel coordinates by means of an intrinsic matrix, written in matrix form and solving for the world coordinates, can be obtained as follows:

(10) XwYw=u−cxR31−fxR11u−cxR32−fxR12v−cyR31−fyR21v−cyR32−fyR22−1fxtx−u−cxtzfyty−v−cytz

Finally, it is combined with the extrinsic matrix to unify to the camera coordinate system. According to the above steps, we obtain the 3D coordinate data of multiple points in the camera coordinate system. Specifically, let the 3D point set be $\left\{\left(x_{i},y_{i},z_{i}\right)\right\}$; first, center the data, i.e., subtract the mean value of the data set from each point, so that the processed data are symmetrically distributed around the origin, and the centered data are $\left\{\left(x_{i}^{\prime },y_{i}^{\prime },z_{i}^{\prime }\right)\right\}$, and we would like to fit a planar equation ax+by+cz+d=0. The problem can be transformed into solving the least squares problem, i.e.,:

(11) $$min\sum_{i=1}^{N}\;{\left(ax_{i}^{\prime }+by_{i}^{\prime }+cz_{i}^{\prime }\right)}^{2}$$

To solve the above problem, the matrix is constructed as the following matrix:

(12) $$A=\left[\begin{array}{ccc} x_{1}^{\prime } & y_{1}^{\prime } & z_{1}^{\prime }\\ x_{2}^{\prime } & y_{2}^{\prime } & z_{2}^{\prime }\\ \vdots & \vdots & \vdots \\ x_{N}^{\prime } & y_{N}^{\prime } & z_{N}^{\prime } \end{array}\right]$$

The matrix A is decomposed into $A=U\Sigma V^{T}$ using SVD, and by SVD decomposition, and the problem of minimizing the error can be converted into finding a right singular vector n=[a, b, c] corresponding to the smallest singular value in $V^{T}$. This vector will correspond to the plane parameters a, b, c. Finally, it is sufficient to bring back the original equation to find the distance d from the origin to the optical plane.

For the rotational axis fitting problem, input a set of rotational axis directions $a_{i},i=1,2,\ldots ,N$. We can find the rotational axis orientation parameter p that minimizes the sum of squares of all residuals, and thus find an optimal rotational axis orientation parameter:

(13) $$p^{*}=\arg\underset{p}{min}\;\sum_{i=1}^{N}\;{∥a_{i}-\frac{p}{∥p∥}∥}^{2}$$

Additionally, a circular trajectory fitting model is introduced to determine the rotation center. The camera center projection points at different rotation angles, denoted as $\left(x_{i},y_{i}\right)$, are used to transform the circle equations into a linear system:

(14) $$\left[\begin{array}{ccc} 2x_{1} & 2y_{1} & 1\\ \vdots & \vdots & \vdots \\ 2x_{n} & 2y_{n} & 1 \end{array}\right]\left[\begin{array}{c} a\\ b\\ c \end{array}\right]=\left[\begin{array}{c} x_{1}^{2}+y_{1}^{2}\\ \vdots \\ x_{n}^{2}+y_{n}^{2} \end{array}\right]$$

This system is solved using the least squares method to obtain the circle center.

## 4. Centerline Extraction by ALWC Method with Five-Step Preprocessing

Underwater laser stripes are affected by various factors such as water turbidity, multiple scattering, background light interference, and uneven target surface reflectivity, which often cause edge blurring, discontinuities, and low signal-to-noise ratio (SNR) in captured images. Traditional centerline extraction methods are computationally efficient and structurally simple, yet they tend to be unstable under high-noise and complex background conditions, resulting in laser stripe drift or center deviation.

To address these challenges in low turbidity underwater environments, this chapter proposes a five-step preprocessing ALWC method for robust laser stripe centerline extraction.

### 4.1. Centerline Image Preprocessing

To enhance detectability and continuity of laser stripes in underwater images, a five-stage preprocessing pipeline is implemented prior to centerline extraction. This pipeline sequentially applies Gaussian filtering, bilateral filtering, top-hat background removal, contrast-limited adaptive histogram equalization (CLAHE) for local contrast enhancement, and Laplacian edge sharpening. The design aims to maximize the removal of turbidity-induced background noise, scattering interference, and stripe blurring, thereby improving the image’s SNR and local contrast.

1. A Gaussian filter is first applied to suppress high-frequency noise introduced by sensor acquisition and scattering in water. This step provides an initial smoothing effect, ensuring that random noise fluctuations are reduced without excessively blurring the stripe edges.
2. To further refine noise suppression while preserving structural details, bilateral filtering is employed. Unlike purely linear smoothing, this edge-preserving filter reduces background noise while maintaining the sharp intensity transition along the stripe boundary.
3. Uneven illumination and background light interference are addressed using morphological top-hat transformation. This step effectively removes slowly varying background components and highlights the stripe as a distinct feature.
4. To enhance local contrast and mitigate the effect of low SNR, CLAHE is performed. This adaptive contrast enhancement improves the stripe’s visibility in darker or low-reflectivity regions of the image while avoiding over-amplification of noise through the contrast-limiting mechanism.
5. Finally, Laplacian-based sharpening is applied to reinforce the stripe’s edge definition. This operation increases the local intensity gradient around the stripe boundaries, ensuring higher accuracy in the subsequent ALWC-based centerline localization.

### 4.2. Adaptive Locally Weighted Centroid Extraction Algorithm

**Figure 9:**
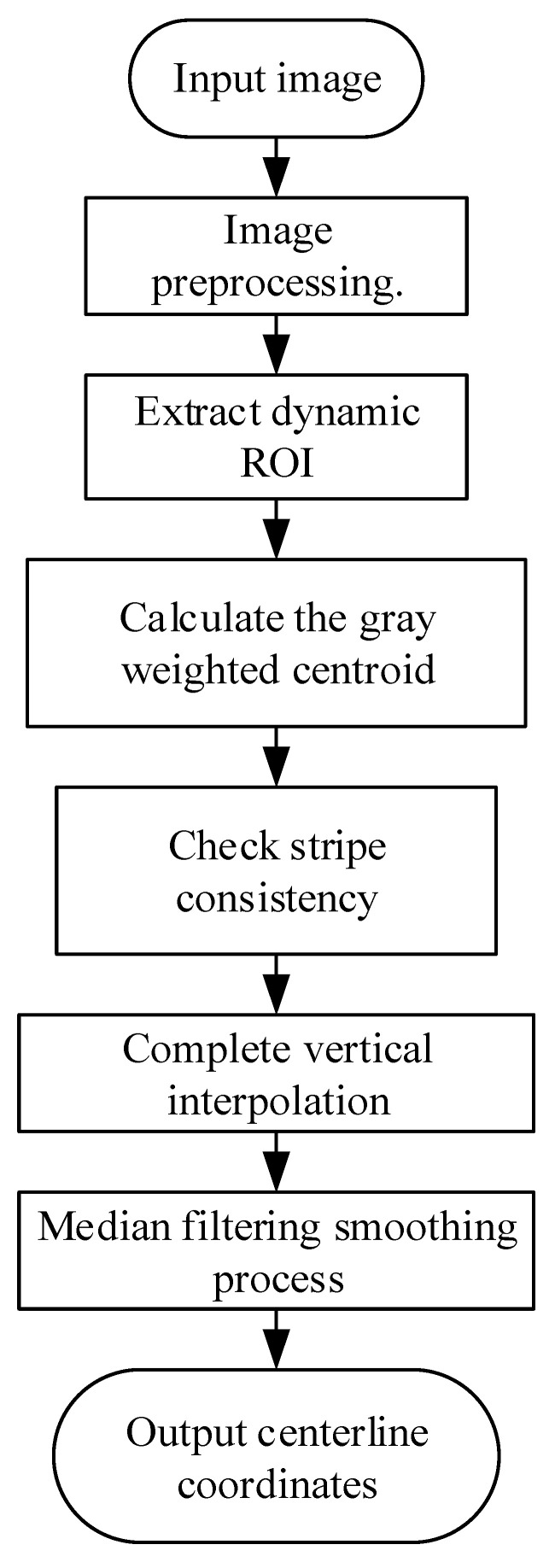
Flowchart of centerline extraction algorithm.

Figure 9 illustrates the main steps of the ALWC extraction algorithm:

To reduce unnecessary processing of the entire image, a dynamic region of interest (ROI) extraction method based on luminance distribution is first employed. Each image row is scanned to identify potential bright regions corresponding to laser stripes. Pixels with luminance values exceeding μ+λ⋅σ are retained, where μ and σ denote the mean and standard deviation of pixel intensities in the row, respectively, and λ is an empirically determined threshold. During scanning, the neighborhood size and weighting coefficients are adaptively adjusted according to the local width and intensity of the laser stripe.

In the dynamic ROI region, for the ROI pixel within row v, take the pixel point with the first n pixel values, and record its column coordinate as $u_{i}$ and brightness as $I_{i}$, then the center of the laser bar in that row is

(15) $$u_{c}\left(v\right)=\frac{\sum_{i-1}^{n}\;I_{i}\cdot u_{i}}{\sum_{i-1}^{n}\;I_{i}}$$

To enhance stability, n is adaptively varied based on the total intensity of the columns, allowing more pixels in brighter regions to participate. Additionally, a low-intensity rejection mechanism excludes pixels with brightness below the 10th percentile of the entire ROI, mitigating noise influence.

Post-extraction, intermittent, spurious, or missing points may arise from uneven reflections, image defects, or underwater disturbances, causing discontinuities detrimental to subsequent 3D reconstruction. To address this, interpolation is introduced. Bidirectional residuals are computed for extracted points as

(16) $$\Delta_{u}\left(v\right)=\left|u_{c}\left(v\right)-\frac{u_{c}\left(v-1\right)+u_{c}\left(v+1\right)}{2}\right|$$

The point is labeled as an anomaly if $\Delta_{u}\left(v\right)>\tau_{u}$ where $\tau_{u}$ is the tolerance threshold between column coordinates and luminance, defined as 2.5 times the median absolute deviation (MAD).

For the row $v_{j}$ labeled as missing, if there are valid points $\left(v_{1},u_{c}\left(v_{1}\right)\right),\left(v_{2},u_{c}\left(v_{2}\right)\right)$ on the upper and lower sides, then linear interpolation is used to estimate the missing values:

(17) $$u_{c}\left(v_{j}\right)=u_{c}\left(v_{1}\right)+\frac{v_{j}-v_{1}}{v_{2}-v_{1}}\cdot \left[u_{c}\left(v_{2}\right)-u_{c}\left(v_{1}\right)\right]$$

Finally, we smooth the centerline trajectory by median filtering (1D median filter): let the sequence of centerline coordinates be $\left\{u_{c}(v)\right\}$, and at index i, the sliding window length is 2k+1; the output of median filtering is

(18) $$u_{c}^{\prime }\left(v\right)=median\left(u_{c}\left(v-k\right),\ldots ,u_{c}\left(v\right),\ldots ,u_{c}\left(v+k\right)\right)$$

Median filtering effectively suppresses isolated spikes while preserving the overall centerline shape.

## 5. Results and Analysis

### 5.1. Calibration Results Validation

**Figure 10:**
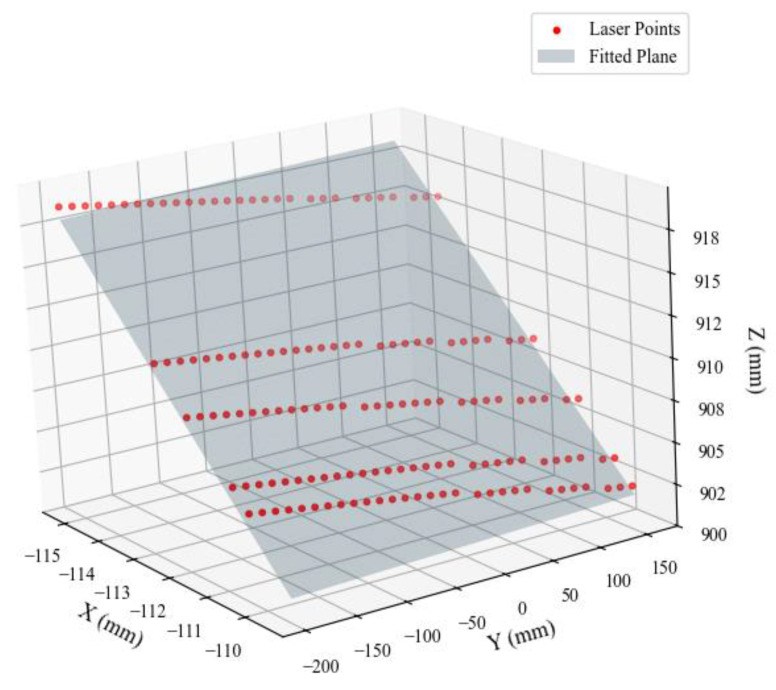
Fitting result of the laser plane in camera coordinate system.

To verify the accuracy of the laser plane fitting in the proposed joint calibration method, Figure 10 presents the 3D distribution of laser centerline points in the camera coordinate system alongside the corresponding fitted plane. The figure shows that the laser points are well distributed and closely aligned with the fitted plane overall. It should be noted that the parameters of the fitted laser plane may change slightly over time due to factors such as mechanical vibrations, temperature variations, or minor shifts in the setup. For practical applications, periodic recalibration is recommended to maintain accuracy. Experimentally, the general equation of the optical plane is obtained as follows:

(19) −0.943923x+0.000497y−0.330164z+194.355111=0

**Figure 11:**
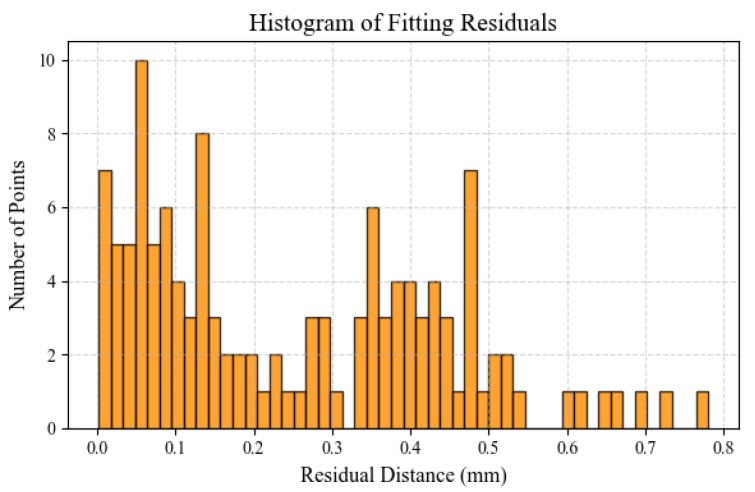
Histogram of laser plane fitting residuals.

The histogram of residuals in Figure 11 further shows that the vast majority of fitting residuals are below 0.5 mm, with very few points exceeding 0.6 mm error. To evaluate the fit quality, the perpendicular distances from all points to the fitted plane were calculated, and the root-mean-square error (RMSE) was determined to be 0.318 mm. This indicates that the proposed method achieves high precision and consistency in the fitting process.

In this study, the relative rotations between neighboring frame pairs are computed based on their extrinsic matrices, from which the rotation axis direction vectors are extracted. The global rotation axis direction is then estimated by fitting multiple frames with the least squares method. The rotation center position is further estimated and validated through the camera trajectory points on the circular path. The fitted unit vector of the rotation axis is $n_{c}=[-0.0027,-0.99999,-0.00399{]}^{T}$.

This vector is nearly parallel to the *Y*-axis of the coordinate system, indicating that the rotation axis of the platform is primarily aligned with the spatial Y-direction, consistent with the design of the experimental setup. To clarify the axis orientation of the camera coordinate system (see Figure 5a): the *X*-axis points horizontally along the image plane, the *Y*-axis points vertically downward, and the *Z*-axis points forward along the optical axis (away from the camera). The residual root-mean-square error (RMSE) between the relative rotation axes of individual frames and the globally fitted rotation axis is 0.89°, demonstrating high consistency of the rotation direction across frames.

Furthermore, the positions of the camera optical centers were computed from five extrinsic matrices, representing the camera’s location in the world coordinate system at each measurement instance. To characterize the camera’s rotational motion, these 3D points were projected onto a plane perpendicular to the estimated rotation axis. A least-squares circle fitting was then performed on the projected points to determine the circle center by minimizing the Euclidean distances from the points to the circle. The fitted circle radius was 218.8 mm. For validation, the approximate radius of the camera’s circular trajectory was measured manually by determining the distance from the camera optical center to the rotation axis using calipers, yielding a value of 223.4 mm. The absolute error between the fitted and measured radius was 4.6 mm, corresponding to a relative error of 2.1%. The close agreement between the fitted and measured values, together with the small fitting residual, demonstrates the high accuracy and reliability of the proposed calibration procedure, confirming the validity of the assumed circular motion trajectory.

### 5.2. Centerline Extraction Results and Analysis

To simulate typical weak underwater visual conditions, kaolin particle suspension is used as the experimental medium with turbidity controlled at approximately 6 NTU, intensifying scattering, attenuation, and background interference. The scanning distance is fixed at 1.0 m.

**Figure 12:**
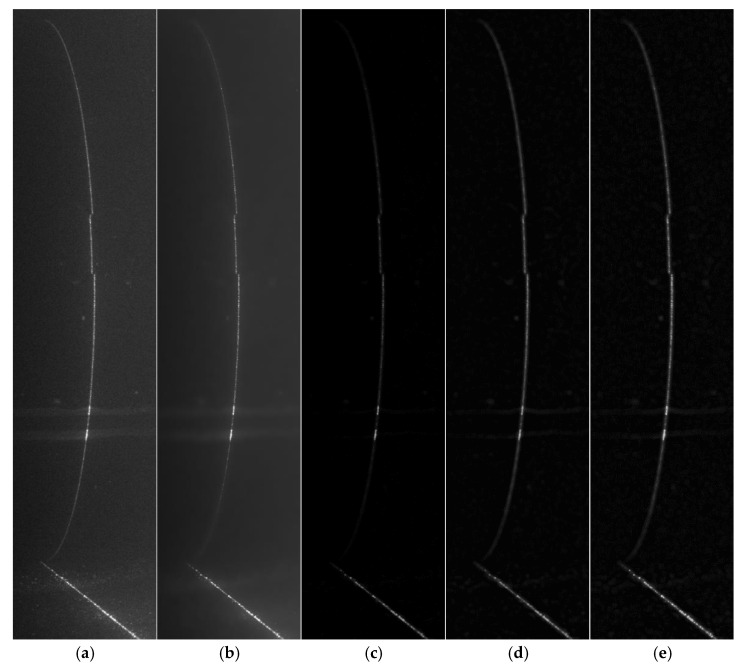
Five-step preprocessing results: (**a**) original grayscale image; (**b**) Gaussian and bilateral filtering; (**c**) top-hat after background removal; (**d**) CLAHE-enhanced image; (**e**) Laplacian sharpening result.

Figure 12 presents the comparative results of images at each stage of the preprocessing pipeline under 6 NTU turbidity. As illustrated, Figure 12a is the original grayscale image, which suffers from low contrast and obvious background noise due to underwater scattering; Figure 12b shows the image after Gaussian filtering and bilateral filtering, where high-frequency noise is effectively suppressed while the edges of laser stripes are preserved; Figure 12c displays the result of top-hat morphological transformation, which eliminates dark background interference and highlights the laser stripe regions; Figure 12d presents the image enhanced by CLAHE, a process that significantly improves the local contrast of dim stripes; and Figure 12e shows the final image after Laplacian sharpening and image fusion, further clarifying the edges of the laser stripes. Experimental analyses reveal that the proposed preprocessing pipeline yields a 3.73 dB improvement in global image SNR and a 76% enhancement in local contrast.

With the five-step preprocessing pipeline effectively enhancing image quality, the subsequent analysis focuses on extracting laser stripe centerlines and comparing the performance of diverse algorithms. To systematically evaluate the applicability of different centerline extraction methods for underwater laser stripes, several representative algorithms are selected for comparative experiments, including the skeleton thinning method, gray centroid method, Steger algorithm, and curve fitting method.

**Figure 13:**
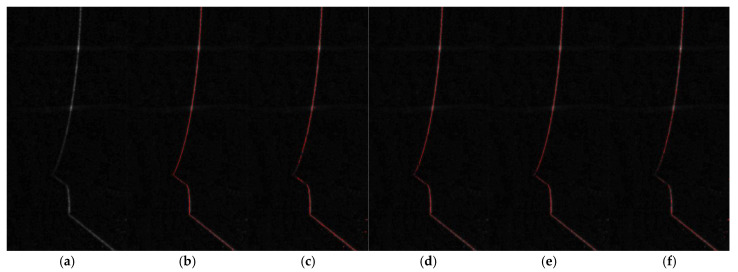
Comparison of centerline extraction results from five algorithms: (**a**) preprocessed original grayscale image; (**b**) centerline extracted by ALWC algorithm; (**c**) centerline extracted by skeleton thinning method; (**d**) centerline extracted by gray centroid method; (**e**) centerline extracted by Steger algorithm; (**f**) centerline extracted by curve fitting method.

Figure 13 presents the visual comparison of the preprocessed original image and centerline extraction results from five algorithms under 6 NTU turbidity, directly reflecting the differences in extraction quality and adaptability of each method to underwater laser stripes. The ALWC algorithm generates a continuous, smooth centerline with no visible breaks along the entire stripe, thanks to its integration of dynamic ROI extraction, stripe consistency detection, and vertical interpolation that compensates for weak or discontinuous regions in underwater stripes.

Table 1 quantifies the performance of the five centerline extraction algorithms under 6 NTU turbidity, focusing on four core metrics—pixel-level root-mean-square error (positioning accuracy), integrity (stripe coverage capability), continuity (centerline smoothness), and efficiency.

Experimental results demonstrate that the proposed ALWC algorithm exhibits high robustness and accuracy in low-turbidity underwater environments. Compared with conventional methods, it maintains stable extraction of continuous and smooth centerlines even under challenging conditions such as partially broken laser stripes and strong brightness fluctuations. Through the integration of dynamic ROI extraction, stripe consistency detection, and vertical interpolation, the algorithm effectively suppresses noise, compensates weak or missing regions.

In terms of integrity and continuity, the ALWC algorithm demonstrates clear superiority. The stripe consistency detection module allows the method to retain weak-signal or discontinuous stripe segments that conventional methods often discard, ensuring nearly complete stripe coverage. At the same time, its real-time deviation correction mechanism prevents abrupt position jumps caused by false pixels, yielding smooth and uninterrupted centerline trajectories.

Furthermore, the algorithm achieves a reasonable balance between accuracy and computational efficiency. While its multi-module design introduces slightly higher computational demand compared with the simplest thinning-based approaches, it remains more efficient than model-heavy algorithms such as the Steger method. Overall, the results validate the rationality of its design and confirm that the ALWC algorithm provides a reliable and efficient solution for underwater laser stripe extraction in low-turbidity conditions.

### 5.3. Underwater 3D Reconstruction Results and Analysis

**Figure 14:**
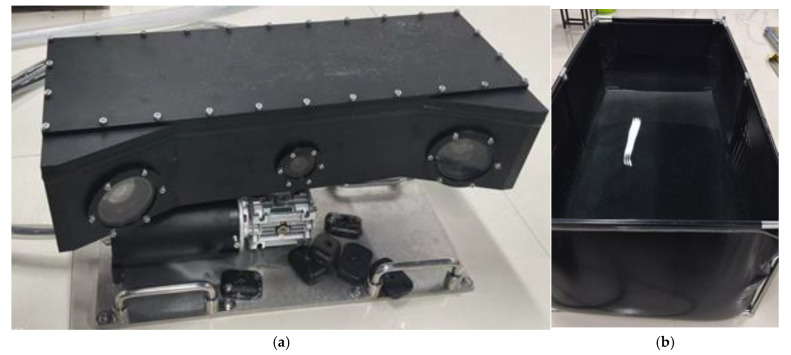
Experimental setup: (**a**) underwater laser scanner prototype; (**b**) experimental pool.

To validate the applicability and accuracy of the proposed calibration method and centerline extraction algorithm under varying water conditions, a series of underwater 3D reconstruction experiments were conducted. Two representative scenarios were considered: a clear water environment (approximately 0.5 NTU) and a manually prepared low turbidity environment (approximately 6 NTU). Kaolin suspensions simulated different turbidity levels, while a professional portable turbidimeter was employed for precise mixing and real-time monitoring to ensure data consistency and reproducibility. Target objects were positioned within a scanning range of about 1 m to evaluate the system’s spatial resolution and reconstruction accuracy under distinct visibility conditions. The underwater laser scanning prototype and experimental setup are shown in Figure 14.

#### 5.3.1. Resolution Evaluation

**Figure 15:**
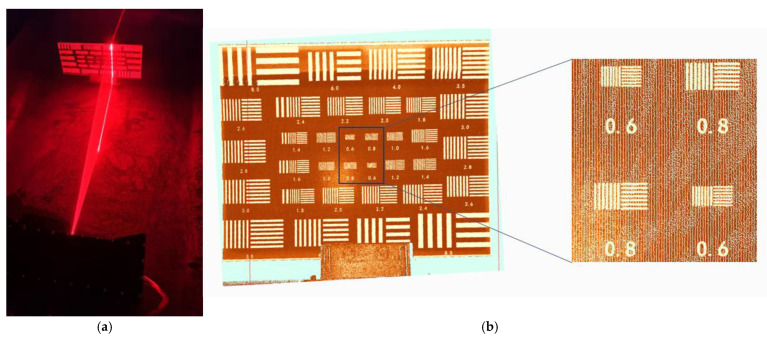
Scanning the resolution plate at a distance of one meter in clear water: (**a**) experimental procedure; (**b**) laser 3D scanning results.

**Figure 16:**
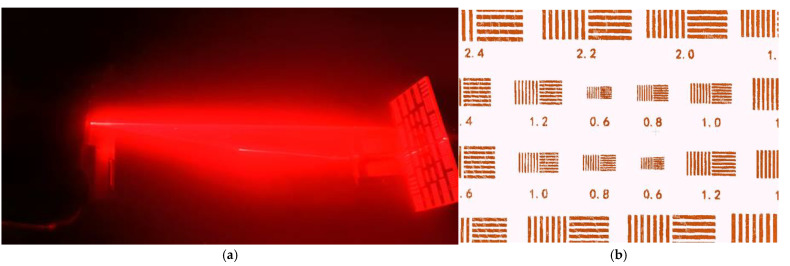
Scanning a resolution plate at a distance of one meter in 6 NTU of turbid water: (**a**) experimental procedure; (**b**) laser 3D scanning results.

Resolution evaluation involved scanning and modeling a standard resolution test plate in clear water and 6 NTU turbid water. Under clear water conditions, the system consistently resolved structures with a minimum line pair spacing above 0.6 mm at 1 m distance. In 6 NTU turbid water, the system maintained a resolution of approximately 0.8 mm despite reduced optical contrast. Figure 15 and Figure 16 present point cloud results for the resolution plates under the respective conditions, both clearly resolving the engraved standard structures, demonstrating fine spatial detail resolution.

#### 5.3.2. Simulation of Pipeline Defect Reconstruction

**Figure 17:**
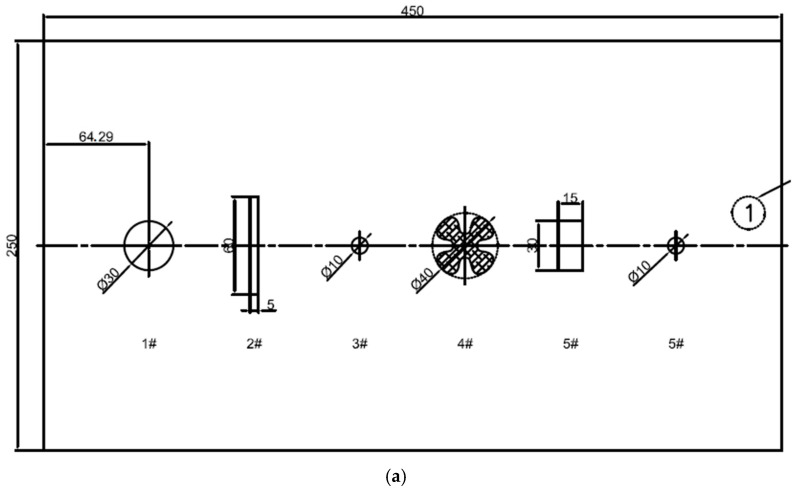

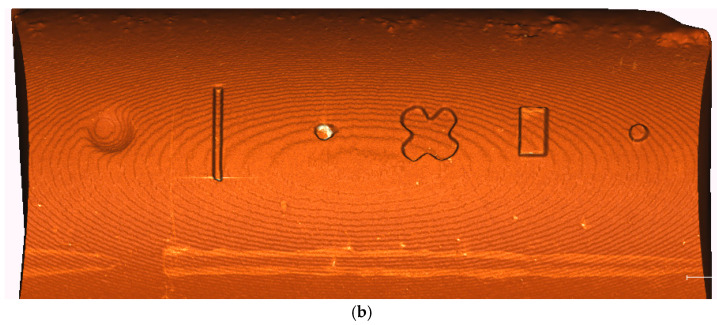
Group 1 simulates pipeline defects: (**a**) dimensional drawings (unit: mm); (**b**) laser scanning 3D reconstruction results.

**Figure 18:**
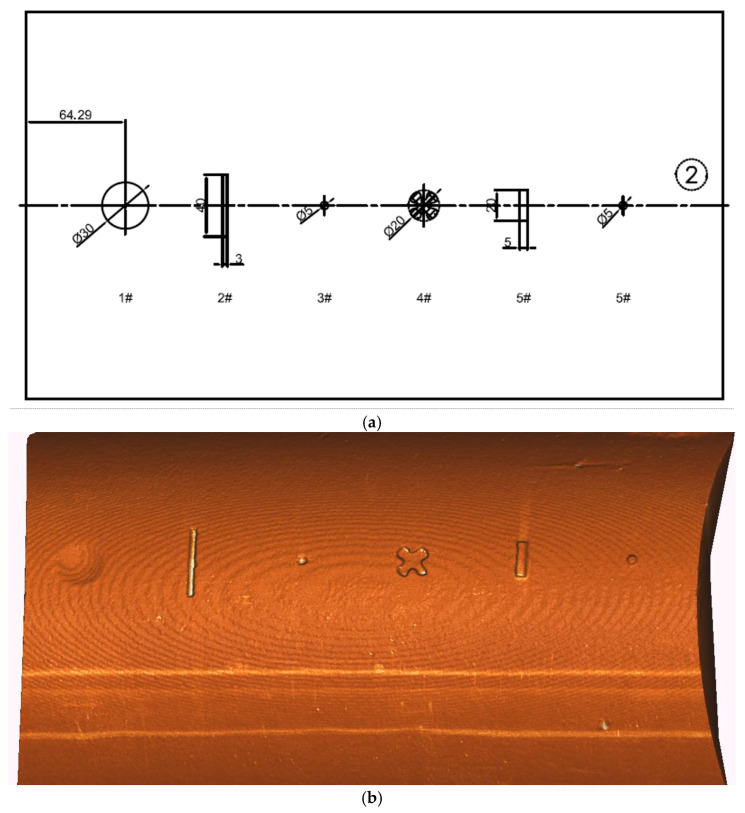
Group 2 simulates pipeline defects: (**a**) dimensional drawings (unit: mm); (**b**) laser scanning 3D reconstruction results.

To further assess 3D reconstruction performance and defect detection under 6 NTU turbidity, a simulated pipeline surface featuring defects such as dents, scratches, small holes, bumps, and grooves was fabricated (Figure 17a and Figure 18a). Geometric feature depths/heights for the first and second groups of defects were 4–5 mm and 2–3 mm, respectively.

At a scanning distance of 1 m under 6 NTU turbidity, the system successfully reconstructed the entire pipeline surface. The reconstructed point clouds (Figure 17b and Figure 18b) clearly delineate defect boundaries and depth contours, indicating effective detection and recovery of fine structures. To quantitatively evaluate reconstruction accuracy, geometric parameters of various defects were extracted and compared with measured dimensions. Table 2 and Table 3 summarize the dimensional error statistics.

Table 2 and Table 3 summarize the three-dimensional reconstruction dimensional errors for the two groups of defects measured under underwater low turbidity (6 NTU) conditions. Each defect type was scanned and measured independently 10 times, with standard deviations maintained within the range of 0.3–0.6 mm. The “average reconstructed size” reported in the tables refers to the mean value of these 10 independent measurements for each defect, representing the typical reconstructed dimension in underwater low turbidity conditions. Experimental outcomes indicate that relative errors for medium-sized defects in both groups were below 5%, demonstrating the system’s reliable capability in scale restoration. For smaller defects, despite some error amplification caused by complex light scattering, the relative errors were predominantly controlled within 10%, confirming the system’s high precision in reconstructing micro-scale structures.

### 5.4. Limitations and Future Work

While the proposed system demonstrates high reconstruction accuracy in low-turbidity conditions (≤6 NTU), its performance is expected to deteriorate under highly turbid water due to increased scattering and absorption. To mitigate this, the existing polarization camera integrated within the system can be leveraged for polarization-differential imaging. By capturing and combining polarization-resolved images, multiple scattering effects can be effectively suppressed, thereby enhancing laser stripe visibility and improving centerline extraction and 3D reconstruction accuracy in challenging high-turbidity environments. Future work will focus on integrating polarization-based processing with advanced deep learning techniques to further enhance robustness under adverse optical conditions.

## 6. Conclusions

This paper presents a compact and adaptable underwater 3D reconstruction system based on line laser scanning with an active rotational mechanism. The system integrates a high-definition camera, a polarization camera, a line laser, and an embedded control module. A joint calibration framework combining in-air calibration with refraction compensation is employed, together with a five-step adaptive locally weighted centroid algorithm and reflectance modeling to enhance accuracy and robustness under low-turbidity conditions. Moreover, a synchronous feature extraction-based method jointly calibrates the laser plane and rotation axis, reducing error accumulation typical in conventional multi-stage approaches. Experimental results demonstrate millimeter-level reconstruction accuracy in both clear water and 6 NTU turbidity, with reconstruction errors below 5% for medium-sized defects and below 10% for defects smaller than 5 mm, confirming the system’s effectiveness and stability for practical underwater applications.

For enhanced adaptability in complex scenarios, future work will modularize the prototype into four waterproof units—two camera housings, a laser-control module, and a rotational system module—interconnected via sealed cables and connectors to improve maintainability and flexibility. Additionally, for high-turbidity environments, polarization-differential imaging combined with deep learning will be employed to further improve imaging quality and overall system performance.

## Figures and Tables

**Table 1:** Comparison of five centerline extraction algorithms under 6 NTU turbidity.

Algorithm	RMSE (Pixels)	Integrity (%)	Continuity (Pixels)	Efficiency
ALWC Algorithm	0.3203	99.21	1.1672	Moderate
Skeleton Thinning Method	1.4579	90.83	1.445	Fast
Gray Centroid Method	1.9693	98.33	1.6575	Moderately Fast
Steger Algorithm	0.4715	98.33	1.154	Slow
Curve Fitting Method	2.4217	80.67	2.5413	Moderately Slow

**Table 2:** Statistical analysis of reconstructed dimensional errors for Group 1 defects.

Type of Defect	Actual Size (mm)	Average Reconstructed Size (mm)	Absolute Error (mm)	Relative Error (%)	Standard Deviation (mm)
Dents	Φ30.00	29.41	0.59	1.97%	0.37
Scratches	60.00	59.12	0.88	1.47%	0.45
	5.00	4.66	0.34	6.80%	0.28
Small holes	Φ10.00	9.53	0.47	4.70%	0.42
Grooves	Φ40.00	38.86	1.14	2.85%	0.53
Bumps1	30.00	29.25	0.75	2.50%	0.39
	15.00	14.52	0.48	3.20%	0.26
Bumps2	Φ10.00	9.57	0.43	4.30%	0.47

**Table 3:** Statistical analysis of reconstructed dimensional errors for Group 2 defects.

Type of Defect	Actual Size (mm)	Average Reconstructed Size (mm)	Absolute Error (mm)	Relative Error (%)	Standard Deviation (mm)
Dents	Φ30.00	28.84	1.16	3.87%	0.41
Scratches	40.00	39.05	0.95	2.38%	0.52
	3.00	2.75	0.25	8.33%	0.33
Small holes	Φ5.00	4.52	0.48	9.60%	0.46
Grooves	Φ20.00	18.92	1.08	5.40%	0.49
Bumps1	20.00	18.77	1.23	6.15%	0.51
	5.00	4.58	0.42	8.40%	0.37
Bumps2	Φ5.00	4.62	0.38	7.60%	0.58

## Data Availability

The data presented in this study are available on request from the corresponding author.
